# Neuropeptide Y in first-episode schizophrenia: is there any sex differences in the pathogeneses of schizophrenia?

**DOI:** 10.3389/fpsyt.2024.1514475

**Published:** 2024-12-03

**Authors:** Jia-Qi Song, Wen Xin, Jian-Jin Yu, Qing Zhao, Hong-Na Li, Da-Chun Chen

**Affiliations:** ^1^ Departments of General Psychiatry, Beijing Huilongguan Hospital, Beijing, China; ^2^ Peking University Huilongguan Clinical Medical School, Beijing, China

**Keywords:** neuropeptide Y, schizophrenia, sex difference, risperidone, PANSS, cohort study

## Abstract

**Objective:**

This study investigated relationships between Neuropeptide Y levels and severity of psychiatric symptoms in first-episode schizophrenia patients, and explore the sexual heterogeneity in them.

**Methods:**

We recruited 115 first-episode schizophrenia patients and 58 matched healthy controls, and measured serum Neuropeptide Y levels of them at baseline and again after 10 weeks of risperidone treatment in patient group. Patients were also evaluated with the Positive and Negative Symptoms Scale (PANSS) to reveal the severity of symptoms.

**Results:**

95 patients completed the whole experiment. We find that mean Neuropeptide Y levels at baseline were significantly higher in patients than in controls (p<0.001), no matter in males or females. In males, there are positive correlations between Neuropeptide Y levels and PANSS scores at baseline (p<0.01), and between the change of them (p<0.05). However, we do not find these correlations in female patients. Furthermore, the interaction terms of NPY × sex were highly significant taking PANSS as dependent variable(p<0.001).

**Conclusion:**

Neuropeptide Y plays a significant role in the pathogenesis of schizophrenia. In male patients, Neuropeptide Y is positively correlated with the severity of symptoms, while this correlation is not found in females. Continued efforts are needed to determine the sexual dimorphism in pathogeneses of schizophrenia.

## Introduction

Schizophrenia is a chronic psychiatric disease with high disability rate. The manifestations of schizophrenia include positive symptoms such as delusion, hallucination, disorganized speech and behavior, and negative symptoms containing social withdrawal, emotional dullness and avolition, while cognitive function is also affected such as memory, executive function, and processing speed (1). In fact, schizophrenia affects about 1% of the global population and is one of the top ten disabling diseases (2). However, with countless patients and their families suffering from schizophrenia, we still have a long way in exploring the pathogenesis of schizophrenia. At present, it is generally believed that the etiology of schizophrenia is related to various factors, which involve genes, neurodevelopment, environmental factors together with social and psychological factors. There are also many hypotheses about the pathogenesis. The mainstream is the neurobiological hypothesis, which suggesting that the schizophrenia mainly originates from the dysfunction of neurotransmitters in the brain, such as dopamine, serotonin, glutamatergic and other neurotransmitters, and this hypothesis has also been confirmed by the evidence of psychopharmacology.

Neuropeptide Y(NPY) was first discovered by Tatemoto in 1982 (3), which is a polypeptide chain composed of 36 amino acids with tyrosine amide group at the C-termina (4). NPY acts on 6 receptors, namely Y1, Y2, Y3, Y4, Y5 and Y6. However, Y3 receptor is not expressed in mammals, while Y6 receptor is not expressed in primates (5). These receptors are distributed in the cortex, amygdala, hypothalamus, hippocampus and striatum in the central nervous system, and are also widely distributed in the heart, kidney, liver, pancreas and intestine (6). Among them, Y4 receptor is mainly distributed in the intestine and less in the central nervous system. In other words, the central system mainly distributes Y1, Y2 and Y5 receptors (7). Through these receptors above, NPY can increase food intake, promote energy to be converted into fat, alleviate anxiety and pain, and also affects biological rhythms (8).

Previous studies have suggested that there were associations between Neuropeptide Y and schizophrenia. For example, in the DISC1(disrupted-in-schizophrenia 1) model, which is the candidate gene locus first described in a large Scottish family (9), NPY-immunoreactive neurons expressions in the prefrontal lobes of DISC1-knockout mice were decreased compared to wild type (10). The study on the brain of schizophrenia patients indicated that, compared with healthy controls, the proportion of NPY neurons in the upper layers of cortex was lower while the expression was abnormally increased in the deep white matter (11). Researchers also found reduced expression of NPY in the prefrontal cortex of schizophrenia patients (12). NPY levels of medication-naïve chronic schizophrenia patients was higher than controls (13).

Antipsychotics can alter the expression of NPY. Mice fed with risperidone for 4 weeks showed decreased NPY mRNA levels in the hypothalamus, but no difference was found in NPY levels in cerebrospinal fluid or peripheral blood (14). The olanzapine would raise the NPY expression in the arcuate nucleus of rats (15), and it was possibly through the NPY pathway that betahistine reversed the weight gain induced by olanzapine (16). The plasma concentrations of clozapine (17) and olanzapine (18) are both negatively correlated with NPY levels.

Some studies have reported that sex heterogeneity was found in many aspects of schizophrenia, including the age of onset, psychiatric symptoms, cognitive function, and treatment efficiency (19). For instance, chronic male patients had an earlier onset of schizophrenia and more severe cognitive impairment and negative symptoms (20). It has been suggested that in many clinical, epidemiological, and fundamental researches that oestradiol could have protective effects in schizophrenic psychoses (21). More interestingly, NPY seemed to be different in males and females. Serum NPY levels were higher in women than in men in chronic patients treated with clozapine (22). Previous studies on stress-related diseases also showed that there were different changes in NPY levels between males and females under acute or chronic stress stimuli (23). NPY in mice showed sexual dimorphism under chronic stress. Social isolation would indicate the decreasing of NPY mRNA levels in the hippocampus of females (24). In prefrontal cortex, NPY mRNA levels were elevated under chronic stress in female mice but not in males (25).

The relationship between schizophrenia and stress can be understood from several perspectives: A characteristic of schizophrenia is subcortical hyperdopaminergia, which has been found to be associated with chronic psychological stress (26). Physiological stress, as measured by allostatic load (AL), is elevated in patients with first-episode schizophrenia and shows a direct correlation with the severity of positive symptoms (27).Schizophrenia patients show heightened anticipation and negative affect in response to stress, indicating difficulties in cognitive stress regulation (28). This impaired ability to manage stress, in turn, intensifies their reliance on negative coping strategies, creating a feedback loop that further deteriorates cognitive performance under stress and amplifies negative outcomes (29).

We speculate that there may be a relationship between sex and NPY in schizophrenia, but there is a dearth of studies related to this field, indicating that we need more efforts to make it clear. Because of the restrictions of medical ethics, it is difficult to collect the samples of cerebrospinal fluid. Fortunately, the NPY cerebrospinal fluid/plasma concentration was basically stable at the level of 20%~40% (30), which means the plasma NPY, to some degree, can reflect the NPY level in the central nervous system.

Therefore, the objective of our study was to investigate relationships between the serum NPY levels and severity of psychiatric symptoms in schizophrenia patients, especially to explore the sexual heterogeneity, which would help explore what role NPY may play in schizophrenia.

## Methods

### Study population

We recruited patients with first-episode schizophrenia who had been admitted to our hospital from 2020 to 2022. The inclusion criteria comprised: (1) patients of Han Chinese ethnicity; (2)patients aged between 18 and 45 years; (3) patients diagnosed with schizophrenia according to diagnostic criteria in the Diagnostic and Statistical Manual of Mental Disorders(4th Edition; DSM-IV); and (4) patients with an drug-naïve first-episode schizophrenia defined as a disease duration ≦̸60 months and with either no previous use of antipsychotics or usage of <14 days (31). Exclusion criteria include: (1) substance abuse or dependence; (2) allergic or autoimmune diseases; (3) definite central nervous system; (4) pregnant or lactation; (5) diabetes mellitus or abnormal glycolipids; and (6) other major physical or infectious diseases. Healthy controls from Residents of Changping District, Beijing were recruited through advertisements and information. Previous researches have shown that people with schizophrenia tend to have shorter years of education; therefore, we recruited people with lower levels of education for matching them to our patient group. We also attempted to match other factors as closely as possible; however, due to significant differences in marital status between the patient and control groups, the final included samples still differed in this regard.

All participants were thoroughly examined, including routine physical examination, blood and urine analyses, and imaging examination to rule out a history of persistent infection, allergy, or autoimmune disease. The study was approved by the Institutional Ethical Review Board of Peking Huilongguan Hospital. All participants provided written informed consent, and we collected data in compliance with the Helsinki Declaration.

### Clinical measures

The Positive and Negative Symptoms Scale(PANSS) (32) was applied to evaluate the clinical symptoms. In total, there are 30 items: 7 items from the positive subscale, 7 items from the negative subscale, and 16 items from the general psychopathological symptom subscale. The higher the score, the worse the disease. PANSS was managed by experienced grader who had received the consistency training required to ensure uniformity across the study and the intra-group correlation coefficient ≥ 0.85. PANSS were assessed on days of blood sampling at baseline and 10 weeks after risperidone treatment.

### Neuropeptide Y measurements

Venous blood samples were collected from patients and healthy controls at baseline, and these processes were repeated after 10 weeks therapy in schizophrenia patients. Then, the samples were placed in a K2-EDTA anticoagulation vacuum tube and centrifuged (3000 r/min, 10 minutes). The plasma was separated and frozen at -80°C in a refrigerator for later testing. NPY was determined using an enzyme-linked immunosorbent assay (Andygene ELISA kit). The same technician assessed each sample, unaware of the participants’ clinical status. The sensitivity of the enzyme-linked immunosorbent assay was 8 pg/ml. The coefficients of intra/inter variation were respectively 5% and 7%.

### Statistical analysis

R version 4.2.1 was used for all statistical analysis and data plotting. For demographic data conforming to the normal distribution, they are expressed as mean ± standard deviation; for those that do not conform, they are expressed as median(P25, P75). Two-way t-test was used for continuous variable, and chi-square test was used for categorical variables. Depending on being conform to the normal distribution or not, Spearman’s correlation or Pearson’s correlation analysis was used to evaluate the associations between NPY and PANSS scores in different sexes, and the same analysis were applied to the change of these indexes and subscales. Benjamini-Hochberg Procedure was used to make multiple hypothesis testing correction. For possible correlations in different sexes, generalized linear regression analysis, using the Best-subset regression model, was employed to control for various confounding factors such as age, BMI, marital status, smoking status and education level. Interaction effect models were used to analyze NPY interaction with sex on PANSS. The test was performed at an α level of 0.05.

## Results

### Group comparison of demographic characteristics and NPY levels

**Figure 1:**
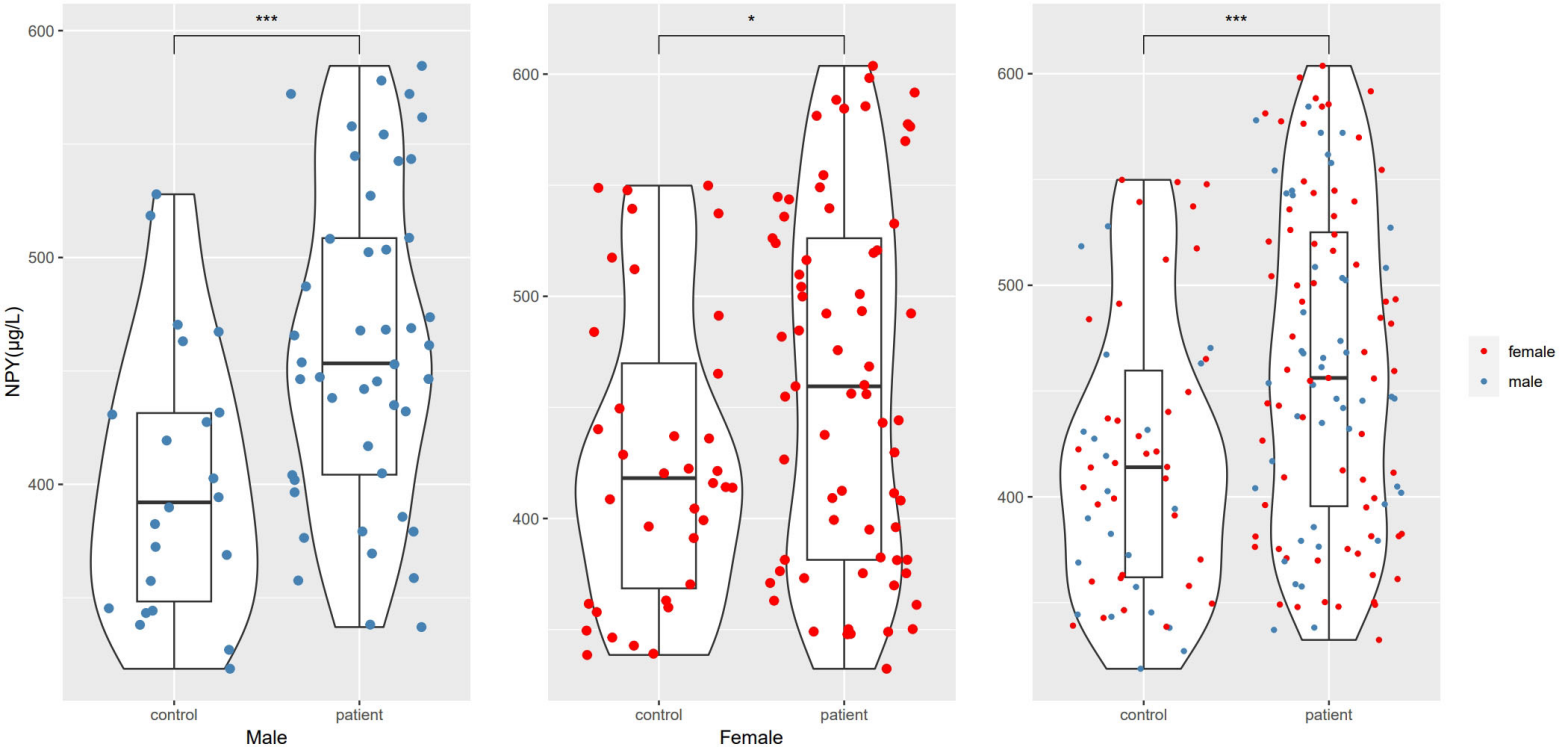
NPY levels differences between schizophrenia and control groups. *represents p<0.05, ***represents p<0.001.

In this study, we enrolled 115 patients with first-episode schizophrenia patients and 58 healthy controls. **Table 1** summarizes the participants’ demographic characteristics. Then among the 115 patients, 20 were excluded because of treatment changes or early patient discharge, and the remaining 95 patients complete the study. In 115 patients and 58 healthy controls at baseline, there was no significant difference in sex, age, years of education, smoking status, BMI and the waist-to-hip ratio between the patient and healthy control groups (p > 0.05). The marriage status in patient group was different from controls (χ^2^ = 11.385, df=3, p=0.010), because there were more unmarried, divorced or widowed people in patients. The serum NPY concentration at baseline was higher in first-episode schizophrenia patients than in controls (t=3.898, df=123.2, p<0.001), no matter in males (t=3.582, df=47.8, p<0.001) or females (t=2.290, df=83.9, p=0.025) (**Figure 1**).

**Table 1:** Demographic characteristics and NPY levels of schizophrenia and control group.

Characteristics		Schizophrenia (n=115)	Control (n=58)	t/χ^2^	df	p value
Gender	Male(%)	46 (40.0%)	22 (37.9%)	0.001	1	0.922
	female(%)	69 (60.0%)	36 (62.1%)			
Age(years)		27.50 ± 8.39	28.22 ± 6.40	-0.635	144.4	0.562
Education(years)		12.89 ± 3.02	12.91 ± 1.55	-0.077	170.9	0.949
Age of onset(years)		25.47 ± 7.9	NA	NA	NA	NA
Disease duration(months)		16 (3, 31)	NA	NA	NA	NA
Smoking status	Yes(%)	99 (86.1%)	46 (79.3%)	0.853	1	0.356
	No(%)	16 (13.9%)	12 (20.7%)			
Marriage status	unmarried(%)	82 (71.3%)	32 (55.2%)	11.385	3	0.010^*^
	married(%)	26 (22.6%)	26 (44.8%)			
	divorced(%)	5 (4.3%)	0 (0.0%)			
	widowed(%)	2 (1.7%)	0 (0.0%)			
Body Mass Index(kg/m^2^)		22.12 ± 4.09	23.25 ± 3.79	-1.798	122.7	0.081
Waist/hip ratio		0.83 ± 0.08	0.84 ± 0.07	-0.560	123.2	0.587
NPY(μg/L)		461.60 ± 76.28	418.33 ± 64.91	3.898	123.2	<0.001^***^

NPY means Neuropeptide Y. ^*^represent p<0.05, ^***^represent p<0.001.

### Changes in pre- and post-treatment NPY levels and PANSS scores in patients

Among the remaining 95 patients completing the study, a paired t-test was used to compare pre- and post-treatment serum NPY levels and PANSS scores. The NPY levels showed no significant difference after 10 weeks of risperidone monotherapy (t=1.410, df=94, p=0.162), no matter in males (t=0.887, df=38, p=0.381) or females (t=1.087, df=55, p=0.282). The PANSS scores decreased significantly (t=17.497, df=94, p<0.001) compared with the scores at baseline.

### Correlation between the NPY and PANSS scores in schizophrenia patients

**Figure 2:**
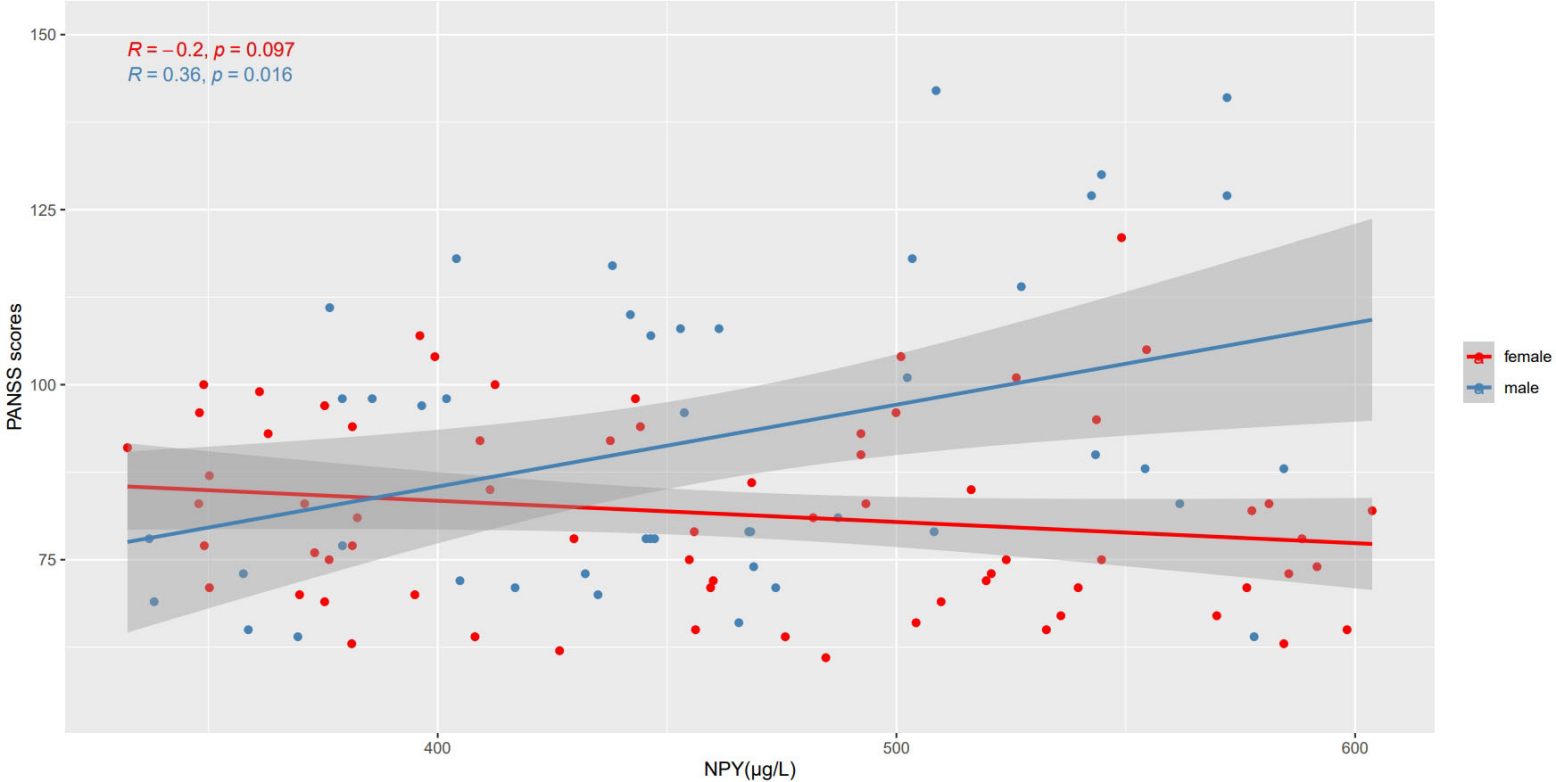
Correlations between NPY levels and PANSS scores in different sexes. At baseline, PANSS scores were positively correlated with NPY concentrations only in male patients, while no correlation was found in females.

We use Spearman’s correlation to evaluate the relationship between NPY and PANSS scores in 115 patients, and respectively in males and females (**Table 2**). At baseline, we found positive correlation between PANSS scores and NPY concentrations only in male patients (r =0.36, p=0.016), and the correlation still exists after Benjamini-Hochberg correction(FDR = 0.048), while no correlation was found in females (r=-0.20, p=0.097), which is illustrated in **Figure 2**. As for subscales, PANSS positive (r=0.38, p=0.011) and general scores (r=0.31, p=0.038) were also positively correlated with serum NPY level in males, and the results in female patients did not show any correlations.

**Table 2:** Correlations between PANSS score and NPY levels during the treatment of risperidone.

	NPY at baseline			NPY change	
	r	p value		r	p value
PANSS	0.01	0.955	PANSS change	0.07	0.496
male	0.36	0.016^*^	male	0.38	0.018^*^
female	-0.20	0.097	female	-0.24	0.070
Positive scores	0.07	0.448	P change	0.10	0.331
male	0.38	0.011^*^	male	0.38	0.018^*^
female	-0.13	0.278	female	-0.17	0.215
Negative scores	-0.02	0.831	N change	-0.14	0.167
male	0.08	0.590	male	-0.03	0.868
female	-0.11	0.349	female	-0.29	0.031^*^
General scores	0.004	0.966	G change	0.16	0.121
male	0.31	0.038^*^	male	0.49	0.002^**^
female	-0.18	0.130	female	-0.19	0.168

^*^represent p<0.05, ^**^represent p<0.01.

**Figure 3:**
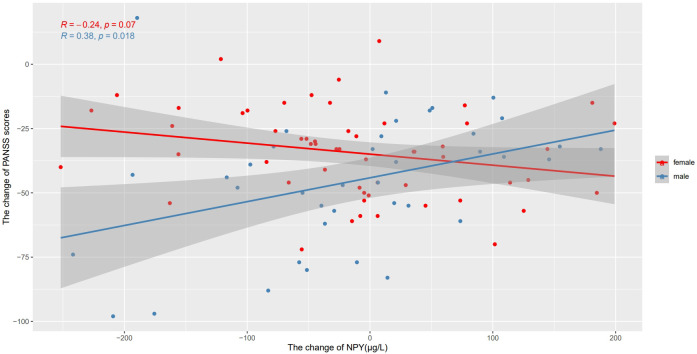
Correlations between the change of NPY levels and the change of PANSS scores after treatment in different sexes. We found the positive correlations between the change of NPY and the change of PANSS in male patients, while no correction was discovered in females.

We also used Pearson’s correlation to explore the relationship between changes in NPY levels and changes in PANSS scores after 10 weeks risperidone treatment (**Table 2**). As shown in **Figure 3**, we found the positive correlations between the change of NPY and the change of PANSS in male patients (r=0.38, p=0.018), while no correction was discovered in females(r=-0.24, p=0.07). However, after FDR correction, the correlation in males only showed a trend (FDR = 0.054). And as expected, we found positive correlations between the change of NPY and the change of PANSS positive scores (r=0.38, p=0.018) along with general scores (r=0.49, p=0.002). What’s more, there was a negative correlation between the changes of NPY levels and PANSS negative scores in females (r=-0.32, p=0.016).

Considering both male and female patients showed possible correlations between PANSS and NPY, we used Best-subset regression model to further exclude the influence of covariates. We took PANSS as the dependent variable, and took NPY, age, BMI, marital status, smoking status and education level as independent variables. Using the generalized linear models selected, in male patients, positive correlations were still found between PANSS scores and NPY levels (t=2.978, p=0.005) and between their changes (t=2.436, p=0.02).

### NPY × sex interaction effect on PANSS as well as their changes

In the interaction effects model which takes PANSS as dependent variable, the interaction terms for NPY × sex were highly significant (t=3.830, p<0.001). And the change of NPY × sex interaction effect on the change of PANSS was also observed (t=3.130, p=0.002). In another word, the relationship between NPY and PANSS can be determined only in males, and the effect of NPY alone was not significant on PANSS (t=-1.156, p=0.250) or its changes (t=-1.514, p=0.133).

## Discussion

To the best of our knowledge, this is the first study to explore the role that NPY plays in first-episode schizophrenia. 1) We found that serum NPY levels were significantly higher in patients with first-episode schizophrenia than in controls. 2) We found that in male patients, there is a positive correlation between the severity of symptoms and the level of NPY, and the change trend of NPY after treatment can reflect the improvement of clinical symptoms. However, the correlations and predictive effects above were not found in female patients.

Serum NPY levels were significantly higher in patients with first-episode schizophrenia. This result also corroborate the findings of prior work that the NPY levels in cerebrospinal fluid of chronic drug-free schizophrenia patients were higher than healthy controls (13). There were also studies that contradicted our findings. In a study in clozapine-treated chronic schizophrenia patients, no difference for NPY was found between the patients and healthy controls (22). However, the included subjects of Wysokiński’s study were all chronic patients treated with clozapine, while it have been proved that clozapine can influence the plasma concentrations of NPY (17). As the first study in first-episode schizophrenia, our study excludes possible interference from antipsychotics, and definitely obtained more reliable results.

The most important finding of our study is that NPY may be used as a predictor of the severity of schizophrenia symptoms. In male patients, NPY is positively correlated with severity of symptoms, and changes in NPY following treatment can reflect improvements in clinical symptoms. Similar findings have been reported by several previous studies. For instance, there was a negative correlation between CSF NPY levels and social function in schizophrenia patients. The higher the NPY at baseline, the worse the social function at the end point of the cohort (33). There were also some studies that contrast with our findings. In a randomized controlled trials of chronic schizophrenia patients with treatment augmented by sarcosine, no relationship between NPY levels and PANSS scores was found, whether at baseline or change (34). After treatment of quetiapine, the NPY level in cerebrospinal fluid (CSF) of schizophrenia patient increased. Further, as defined by more than 20% PANSS reduction, the responder group had higher CSF NPY levels compared to the nonresponders (35). However, these researches included both first-episode and long-term hospitalized patients as subjects, so compared to our study, the results obtained were not reliable enough on account of difficulty in controlling for confounding factors.

We speculate that the relationship of NPY and severity of symptoms may be mediated by dopamine (DA). Previous studies have suggested that NPY can regulate the secretion of dopamine in neurons. For instance, intraventricular injection of NPY agonists can increase the concentration of DA in the nucleus accumbens (36). Y1 and Y5 receptors on the striatum neurons decreased dopamine release, while Y2 receptors played the opposite role (37). A mouse experiment further confirms the idea that NPY indicate changes related to schizophrenia through the dopamine pathway. Peptide tyrosine-tyrosineis 3-36(PYY3-36) is a selective Y2 receptor agonist (38). In mice, administration of PYY3-36 would cause schizophrenia-related behavioral changes, such as impairment in the PPI (paradigm of prepulse inhibition) and water maze test (39). Furthermore, the PPI impairment was reversed by first-generation antipsychotics, but not by clozapine. We speculate that when NPY stimulates the Y2 receptors of neurons, it may cause an abnormal increase in DA, which may explain that the concentration of NPY is proportional to the severity of symptoms. And also, the impairment would be relieved when DA receptors were blocked.

Of course, except for DA, there is also research examining the interactions between neuropeptide Y (NPY) and other neurotransmitters. In mice, NPY has been found to inhibit the activity of serotonergic (5-HT) neurons in the dorsal raphe nucleus (DRN) via activation of Y1 receptors (40). Additionally, NPY increases extracellular glutamate concentration through activation of Y1 receptors in the hippocampus, which has been shown to mitigate seizure-like neuronal activity (41). Furthermore, by modulating 5-HT receptors, NPY may facilitate adaptive coping responses, thereby enhancing resilience in extreme environments (42). However, the results of these studies remain inconclusive, and much work is needed to fully elucidate the underlying mechanisms.

Another significant finding of our study is sexual heterogeneity in the effect of NPY on schizophrenia. The above-mentioned relationships between NPY and severity of symptoms were only found in male patients. Previous experimental studies indicated that the function of Y2 receptor seems to be different between male and female mouse. Male NPY Y2 receptor knockout mice, compared to wild-type, performed better in social interaction, Y-maze and PPI experiments, while the improvements not seen in females (43). In another study of Y1 receptor knockout mice (44), whose subjects were all males, Y1 receptor influenced the acoustic startle response.

We conjecture that the sexual heterogeneity may result from the influence of sexual hormone. The NPY-knockout mice showed sexual dimorphism, and the HPA axis of male mice was abnormally activated when compared to the wild-type (45). Furthermore, NPY-knockout female mice were divided into the ovariectomized group and the normal cycling group, and the ovariectomized also showed metabolic status similar to the male mice (46). It is supposed that NPY can promote the release of GnRH, while NPY itself is regulated by estrogen (47). However, there are many contradictory conclusions about the effect of estrogen on NPY. On the one hand, in NPY knockout mice, the luteinizing hormone(LH) peak stimulated by estrogen was blunted, suggesting that estrogen may increase the expression of NPY (48). On the other hand, there were also studies on the regulation of feeding indicated that estrogen can inhibit the expression of NPY (49). It is also clear that estradiol has a corresponding inhibitory receptor in the regulatory region of the NPY gene (50). The contradictory results of NPY in different brain regions of different sexes may be related to the characteristics of estrogen receptors on neurons (51). As reviewed (23), some studies have identified that aged males had less NPY gene expression compared with young animals in both arcuate nucleus and hypothalamic nuclei, while serum NPY levels would increase with age in females. Therefore, further studies are needed to explore the possible mechanisms in the sexual heterogeneity of NPY.

A few limitations need to be noted regarding the present study. First, only serum NPY levels were measured in this study, though peripheral levels can reflect the concentration in CSF. Evaluation of NPY gene expression and detection of NPY variants could be the next step, which would be more helpful to study the pathogenesis of schizophrenia. Second, after 10 weeks of risperidone treatment, NPY levels did not show significant changes, suggesting a potential lag in NPY response relative to symptom improvement, in future studies, the sample size should be increased, and the follow-up time needs to be prolonged to obtain conclusive results. Additionally, monitoring changes in estrogen and NPY levels across the menstrual cycle could provide further insights into the sex differences.

## Conclusion

NPY plays a significant role in the pathogenesis of schizophrenia. Higher NPY levels were observed in patients with first-episode schizophrenia. Moreover, there is sexual dimorphism of the NPY in schizophrenia. In male patients, NPY levels were positively correlated with the severity of schizophrenia, while this relationship was not observed in female patients. Further researches are needed to ascertain the possible mechanisms underlying this sexual heterogeneity.

## Data Availability

The raw data supporting the conclusions of this article will be made available by the authors, without undue reservation.
